# Emission Flux Measurement Error with a Mobile DOAS System and Application to NO_x_ Flux Observations

**DOI:** 10.3390/s17020231

**Published:** 2017-01-25

**Authors:** Fengcheng Wu, Ang Li, Pinhua Xie, Hao Chen, Zhaokun Hu, Qiong Zhang, Jianguo Liu, Wenqing Liu

**Affiliations:** 1 Key Laboratory of Environmental Optical and Technology, Anhui Institute of Optics and Fine Mechanics, Chinese Academy of Sciences, Hefei 230031, China; fcwu@aiofm.ac.cn (F.W.); hchen@aiofm.ac.cn (H.C.); zkhu@aiofm.ac.cn (Z.H.); zhangqiong@aiofm.ac.cn (Q.Z.); jgliu@aiofm.ac.cn (J.L.); wqliu@aiofm.ac.cn (W.L.); 2 Center for Excellence in Regional Atmospheric Environment, Institute of Urban Environment, Chinese Academy of Sciences, Xiamen 361021, China; 3 School of Environmental Science and Optoeclectronic Technology, University of Science and Technology of China, Hefei 230026, China

**Keywords:** DOAS, spectrophotometer, mobile measurements, emission flux, error, NO_x_

## Abstract

Mobile differential optical absorption spectroscopy (mobile DOAS) is an optical remote sensing method that can rapidly measure trace gas emission flux from air pollution sources (such as power plants, industrial areas, and cities) in real time. Generally, mobile DOAS is influenced by wind, drive velocity, and other factors, especially in the usage of wind field when the emission flux in a mobile DOAS system is observed. This paper presents a detailed error analysis and NOx emission with mobile DOAS system from a power plant in Shijiazhuang city, China. Comparison of the SO_2_ emission flux from mobile DOAS observations with continuous emission monitoring system (CEMS) under different drive speeds and wind fields revealed that the optimal drive velocity is 30–40 km/h, and the wind field at plume height is selected when mobile DOAS observations are performed. In addition, the total errors of SO_2_ and NO_2_ emissions with mobile DOAS measurements are 32% and 30%, respectively, combined with the analysis of the uncertainties of column density, wind field, and drive velocity. Furthermore, the NO_x_ emission of 0.15 ± 0.06 kg/s from the power plant is estimated, which is in good agreement with that from CEMS observations of 0.17 ± 0.07 kg/s. This study has significantly contributed to the measurement of the mobile DOAS system on emission from air pollution sources, thus improving estimation accuracy.

## 1. Introduction

China has been experiencing severe air pollution problem with the booming growth of industrialization and urbanization. The key to alleviate air pollution is the measurement, supervision, and control of its sources. The emission flux of sources is the most important and fundamental data for pollution source assessment. Currently, the primary technique for detecting emission flux is field test method, which calculates organized pollution emission based on observations of exhaust flow rate and air pollutant concentrations. However, the method cannot detect fugitive emissions, especially in area sources. In addition, the model [1,2] is also an alternative method for estimating source emission; however, it is limited to spatial scale and real-time data.

Mobile differential optical absorption spectroscopy (mobile DOAS) [3,4] is a novel optical remote method that can estimate source emission. The technique was developed from fixed-scanning DOAS [5] observation of volcanoes and measurement of point emissions (power plants, oil refineries, etc.) and area (city, industrial areas, etc.) sources with zenith-observation DOAS system mounted on a mobile platform. Johansson et al. [3,4] and Rivera et al. [6] estimated SO_2_, NO_2_, and HCHO emissions from the industrial areas in Mexico, Beijing, and Tula (Mexico) with mobile DOAS from 2008 to 2009. NOx emissions in Mannheim, Ludwigshafen, and New Delhi city were observed by Ibrahim et al. [7] and Shaiganfar et al. [8] in 2010 and 2011 with mobile multi-axis differential optical absorption spectroscopy (mobile MAX-DOAS). From these studies, mobile DOAS has provided a novel and rapid method for source emission measurement. However, one disadvantage of this method is that the observation is affected by actual conditions (such as weather, wind, etc.). Previous studies [6] suggesting either near-surface wind from a ground meteorological station or wind at plume height from a model can be used to estimate emission. However, a detailed discussion on errors from different wind and drive speeds with mobile DOAS observations still lacks. In the current study, one power plant in Shijiazhuang city is selected as a typical point source to discuss the usage of the appropriate wind and drive speed for emission measurement using mobile DOAS. Furthermore, the total emission error of mobile DOAS is estimated based on error analysis for each impact factor of emission measurement.

Meanwhile, as one significant application of mobile DOAS, the NO_x_ emission from a power plant is estimated. NO_x_ is one of the most important trace gases in the atmosphere [9]. It participates in the catalytic formation of ozone (O_3_) in the troposphere, while being a catalyst for O_3_ destruction in the stratosphere [10,11]. NO_x_ sources include nature source and anthropogenic source. The main anthropogenic sources are fossil fuels (coal, oil, etc.) and biomass burning. This study explores the observation of NO_x_ emission from power plants with mobile DOAS preliminary and compares it with the CEMS result, showing a good agreement between the two results.

## 2. Experiment and Principles

### 2.1. Overview of Experiment

**Figure 1:**
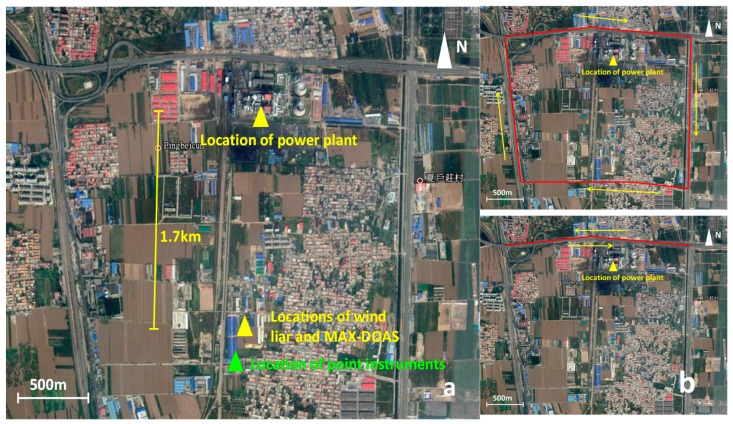
Setup of measurement sites (**a**) and two different driving routes (**b**). Red lines indicate the measurement routes and yellow arrows indicate the driving direction in the (**b**).

A power plant with stack height of 180 m was located at the south of Shijiazhuang city, a typical point source surrounded by farmlands. The construction of the power plant was divided into two stages: The first stage involves the construction of two coal-fired heating units with a total power of 600 MW, which are currently in operation. The second stage plans the construction of another two coal-fired heating units with total power of 700 MW, which is currently being actively promoted. The measurements were conducted from October to November 2011. The entire weather condition was dominated by clouds, and rain occasionally poured from 3 November to 5 November and from 7 November to 8 November for each measurement day, we conducted circular measurements and performed scanning measurements in the north of the power plant, considering good driving conditions for different drive speeds. The entire circle and scanning route was 12 km and 6 km, respectively. We took approximately 30 and 15 min to complete the measurements. The measurement time was from 10:00 to 14:30. Furthermore, one wind LIDAR, MAX-DOAS, and some point instruments detected NO, NO_2_, and O_3_ concentrations established away from the power plant (1.7 km). The setup of the measurement sites and two different driving routes are shown in Figure 1.

### 2.2. Mobile DOAS System

**Figure 2:**
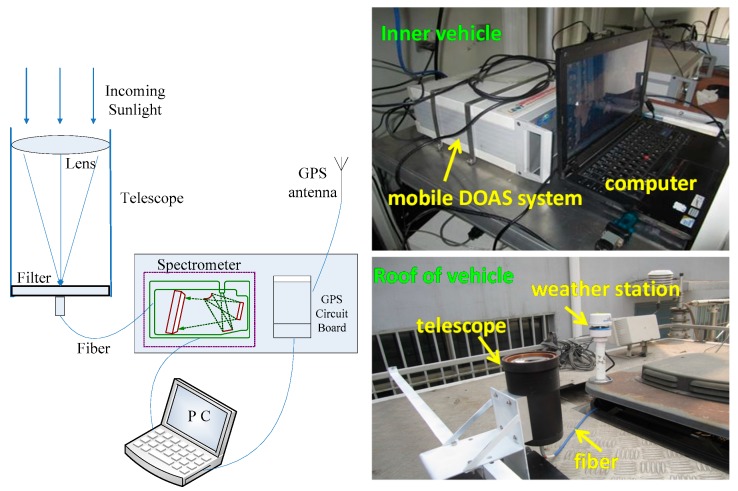
Mobile DOAS system.

The mobile DOAS system was developed at the Anhui Institute of Optics and Fine Mechanics as shown in Figure 2 [12]. The components of the instrument are spectra acquiring unit, data processing unit, and global position system (GPS) module. The spectra acquiring unit includes a zenith observation telescope and optics miniature spectrometer. The telescope collects scattered sunlight in the zenith observation and focuses into a fiber. Light collected by the telescope is transmitted to the spectrometer (HR2000+, Ocean Optics, Dunedin, FL, USA) through the fiber with spectral resolution of 0.6 nm and spectral wavelength range of 290–420 nm. The spectra are then transmitted to the data processing unit to retrieve the concentration in real time through a USB data cable. GPS module is used to record geographic information and drive speed information. The system is also equipped with a miniature weather station mounted on top of the measurement van to obtain meteorological data (wind direction, wind speed, temperature, pressure, etc.). Details of the instrument and its performance are described in our previous study [13].

### 2.3. Principle of Mobile DOAS

#### 2.3.1. Retrieval of Vertical Column Density

The passive DOAS technique has been employed in numerous applications that use sunlight with instruments mounted on various fixed or mobile platforms [14,15,16,17,18,19]. The evaluation procedure is described in this section in relation to our mobile observation. Details of DOAS analysis are presented in Platt and Stutz (2008) [20].

The zenith scattered sunlight *I*_0_ is collected by the mobile DOAS as *I* due to atmospheric extinction. *I* and *I*_0_ agree with the Lambert-Beer law:

(1) $$I=I_{0}\cdot \exp(-\sigma \cdot c\cdot L)$$

where *σ* (unit: cm^2^/molec.) is the cross section of trace gas, *c* (unit: molec./cm^3^) is the concentration, and *L* (unit: cm) is the length of the absorption route. The concentration of trace gas can be evaluated based on the least square fit algorithm using Equation (1). Unknown to the length of absorption route with mobile DOAS observations, the slant column density (*SCD*, unit: molec./cm^2^) can be retrieved using Equation (1). The *SCD* is defined as the trace gas concentration integrated along the effective light path:

(2) $$SCD=\int c(l)\cdot dl=\frac{1}{\sigma }(\frac{I_{0}}{I})$$

The vertical column density (*VCD*) (unit: molecules/cm^2^) can be calculated with the *SCD* and air mass factor (*AMF*, non-dimensional variable) [21] as shown in Equation (3). The *AMF* can be obtained with radiation transfer model and geometric approximation, which depends on the solar zenith angle, wavelength, and elevation angle:

(3) $$VCD=\frac{SCD}{AMF}$$

#### 2.3.2. Estimation of Emission

The emission of source can be detected in combination with the *VCD* and the measurement route, wind speed, and wind direction. For one area of interest, the emission flux is calculated using Equation (4) [3,4,7]:

(4) $$F=\int_{A}div(VCD\cdot \vec{W})\cdot dA=\oint_{S}VCD(\vec{s})\cdot \vec{W}\cdot \vec{n}\cdot d\vec{s}$$

where $\vec{W}$ indicates the average wind vector, *A* indicates the encircled area, $\vec{n}$ indicates the normal vector parallel to the Earth’s surface and orthogonal to the driving direction at the position of the driving route, and $\vec{s}$ is the driving route. For mobile DOAS measurements, we can convert Equation (4) to Equation (5):

(5) $$F=\sum_{i}VCD(s_{i})\cdot \vec{W}\cdot \vec{n}\cdot \Delta s_{i}=\sum_{i}VCD(s_{i})\cdot \vec{W}\cdot \sin(\beta )(s_{i})\cdot \Delta s_{i}$$

where *β* is the angle between the driving direction and wind direction, and Δ*s_i_* is the distance between two successive spectra.

The NOx emission is estimated based on the conversion of NO_2_ emission with two correction factors: chemical transformation factor and lifetime correction factor:

(6) $$F_{NOx}=R\cdot c_{L}\cdot F_{NO_{2}}=R\cdot c_{L}\cdot \sum_{i}VCD_{NO_{2}}(s_{i})\cdot \vec{W}\cdot \sin(\beta )(s_{i})\cdot \Delta s_{i}$$

The chemical transformation factor can be written as R = NO_x_/NO_2_, NO_x_ = NO + NO_2_:

(7) $$R=\frac{NOx}{NO_{2}}=\frac{NO+NO_{2}}{NO_{2}}=1+\frac{NO}{NO_{2}}=1+c_{\tau }$$

*c_τ_* = *NO*/*NO*_2_ is the Leighton ratio [11], which is generally derived from model simulations. In this study, we calculate the value from observation data of point instruments near the power plant.

*c_L_* in Equation (6) is the lifetime correction factor, which is derived using Equation (8):

(8) $$c_{L}=e^{\frac{D/W}{t}}$$

where *D* (unit: meters) is the mean distance from the source to the measurement site of about 2 km during this observation. *t* (unit: second) indicates the NOx lifetime, which depends on photochemical reaction and meteorological condition and is difficult to determine for the specific situation of our measurements. We use an average value of 5 h for the NOx lifetime, which is derived from long-term satellite observations of polluted area in summer and autumn in eastern China [22].

### 2.4. Data Analysis

The VCDs of SO_2_ and NO_2_ are retrieved through the DOAS method as discussed in Section 2.3.1. During our retrieval process, a spectrum is first selected arbitrarily on the upwind path as reference spectrum to determine the concentration distribution trends along the route. The minimum concentration of SO_2_ and NO_2_ along each driving route is then chosen as the Fraunhofer spectra to re-retrieve the measurement spectra.

**Figure 3:**
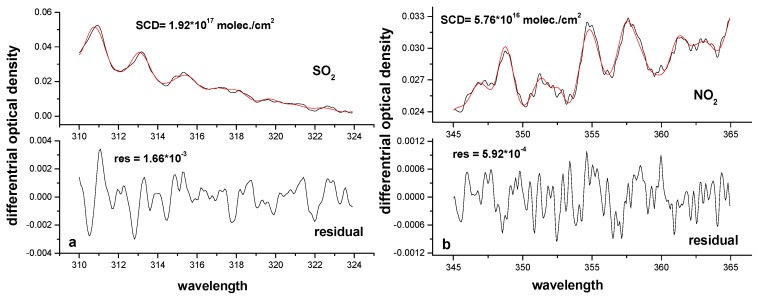
SO_2_ and NO_2_ fitting. The spectra are recorded at the time of 12:35:02 on 16 October 2011. (**a**) SO_2_ retrieval result, SO_2_ SCD = 1.92 × 10^17^ molec./cm^2^, SO_2_ residual = 1.66 × 10^−3^; (**b**) NO_2_ retrieval result, NO_2_ SCD = 5.76 × 10^16^ molec./cm^2^, NO_2_ residual = 5.92 × 10^−4^.

The wavelength range of 310 to 324 nm with three strong absorption peaks is selected for the SO_2_ fit. Absorption cross sections of SO_2_, NO_2_, HCHO, O_3_ (Bogumil et al. [23]), and Ring are included in the fit. The Ring spectrum is generated from the measured Fraunhofer reference spectrum using the DOASIS [24] software. For the analysis of NO_2_, the wavelength range of 345 to 365 nm is selected, and the cross section of O_4_ at 298 K is also included, except for NO_2_, HCHO, O_3_ at 293 K (Bogumil et al. [23]), and Ring spectrum. The wavelength calibration is performed using a highly resolved solar spectrum (Kurucz et al. [25]) convoluted by the instrument’s slit function. The software of WinDOAS [26] is used to evaluate the SO_2_ and NO_2_ SCDs. An example for such a spectral fitting is shown in Figure 3, where the SCD is 1.92 × 10^17^ ± 6.56 × 10^15^ molec./cm^2^ and 5.76 × 10^16^ ± 1.53 × 10^15^ molec./cm^2^ for SO_2_ and NO_2_, respectively. The fit uncertainties of retrieved values from these two spectra for SO_2_ and NO_2_ are about 3.41% and 2.66%, respectively. For all measured spectra, the fit uncertainties are less than 15% for NO_2_ and 20% for SO_2_.

**Figure 4:**
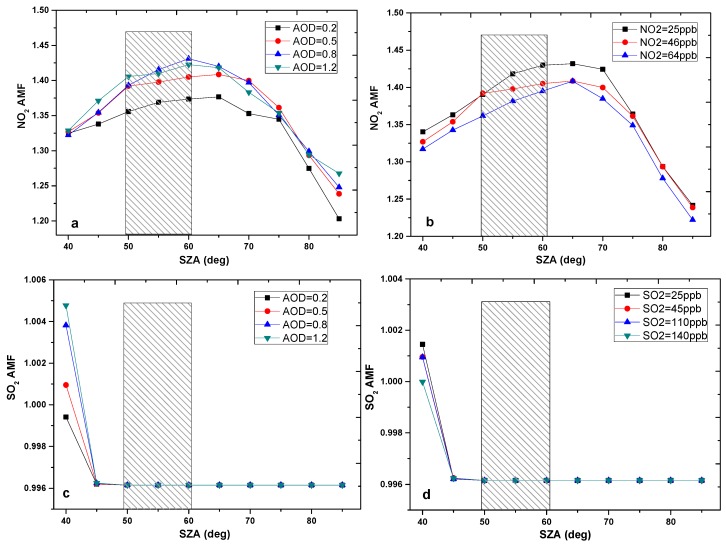
Simulated AMF for different aerosol and trace gas profiles. The rectangle regarding the solar zenith angle (SZA) is the time of mobile DOAS observations. (**a**) NO_2_ AMF simulation in the scenarios of different SZAs and aerosol optical density (0.2, 0.5, 0.8 and 1.2); (**b**) NO_2_ AMF simulation in the scenarios of different SZAs and NO_2_ concentration (25 ppb, 46 ppb and 64 ppb) (**c**) SO_2_ AMF simulation in the scenarios of different SZAs and aerosol optical density (0.2, 0.5, 0.8 and 1.2); (**d**) SO_2_ AMF simulation in the scenarios of different SZAs and SO_2_ concentration (25ppb, 45 ppb, 110 ppb and 140 ppb).

In the previous study, the VCDs of SO_2_ and NO_2_ were derived through the geometric approximation of AMF with mobile DOAS observation. According to the relationship between AMF and elevation angle (*AMF_trop_* ≈ 1/sin(*α*)), the tropospheric AMF is close to 1 due to zenith observation and measurement time at noon. As a result, the retrieved SCD is approximated to VCD. However, *AMF_NO_*_2_ is larger than 1 (Figure 4a,b) and *AMF_SO_*_2_ is approximately equal to 1 (Figure 4c,d), which are retrieved using a radiative transfer model McArtim [27] simulation during the measurement time. In this study, the SO_2_ and NO_2_ VCDs are calculated using the simulated AMF.

AMF strongly depends on NO_2_ and aerosol profiles, which use radiative transfer model simulation. The different scenarios of aerosol, SO_2_, and NO_2_ are set to estimate the uncertainties of SO_2_ and NO_2_ AMF (Figure 4). The height of boundary layer is taken from LIDAR and ceilometer observations away from mobile DOAS (5 km). The concentrations of SO_2_ and NO_2_ are taken from point instruments near the power plant. The average boundary layer is about 1 km, and aerosol optical density (AOD) ranged from 0.2 to 1.2 as observed from the LIDAR and ceilometer. We assumed that the aerosol profiles are given by constant values below the boundary layer height and exponential profiles above for the AMF simulation. The NO_2_ scenarios of 64, 25, and 46 ppb and SO_2_ scenarios of 25, 45, 110, and 140 ppb are set based on the data from point instruments. The NO_2_ and SO_2_ profiles are given with a “box” shape, only aiming at plume observation from the power plant.

NO_2_ AMF uncertainties are about 6%, caused by aerosol and NO_2_ profiles during the measurement period (the solar zenith angle between 50° and 60°) from Figure 4. However, the SO_2_ AMF is free of solar zenith angle, aerosol, and SO_2_ variations. As a result, the uncertainties of SO_2_ AMF can be neglected compared with other errors.

## 3. Analysis of Mobile DOAS Error

### 3.1. Usage of Driving Speed

The selection of driving speed is a key factor for mobile DOAS observations, and the peak value may be lost if the driving speed is too fast. Otherwise, the conversion may be yielded for emission plume resulting in inconsistency between emission and measurement if the speed is too low. Thus, the selection of appropriate speed represented by sampling points during plume observation is important to accurately estimate emission. We have estimated the SO_2_ emission for different samples and compared them with those of CEMS. The optimal sampling is achieved by intercomparison of CEMS and mobile DOAS, and the optimal speed is presented combined with plume width and sampling time.

**Figure 5:**
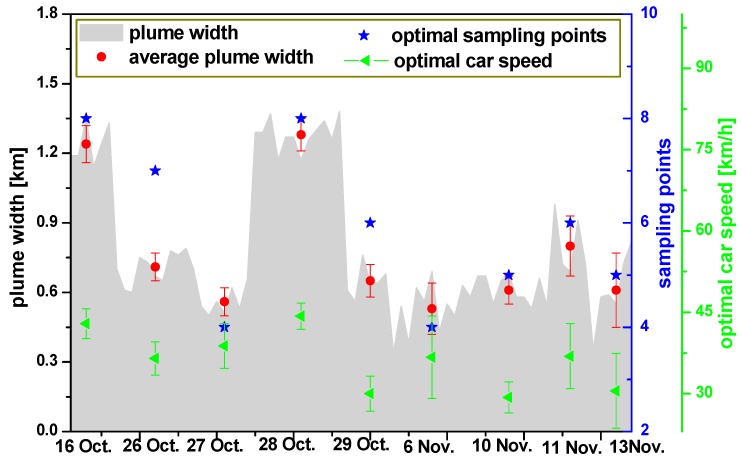
Plume width, average plume width, optimal sampling, and speed.

The average width of plume is calculated based on multiple scanning measurements for different sampling assuming similar weather conditions:

(9) $$L=\frac{\sum_{i=1}^{n}v_{i}\cdot s_{i}\cdot \Delta t_{i}}{n}$$

where *n* indicates the number of times of all scanning measurements, *v_i_* is the driving speed for each scanning measurement, and *s_i_* indicates the number of sampling for each scanning measurement. The average plume width, optimal sampling, and optimal speed are shown in Figure 5. The maximum and minimum plume width is 1.28 ± 0.07 km and 0.53 ± 0.11 km, respectively, during the measurement period. The intercomparison of the results between CEMS and mobile DOAS and the sampling with the minimum difference, which is the optimal sampling, are shown in Figure 5. The optimal driving speed of 36.21 ± 5.44 km/h is calculated based on the average plume width, optimal sampling, and sampling time.

### 3.2. Usage of Wind

Wind information is one of the largest error sources in emission estimation. The question on the usage of ground-based wind or plume-height wind has not been discussed in detail in previous studies. As shown in Figure 1, one wind LIDAR is set up near the power plant to obtain the vertical wind data, particularly the wind data at 200 m height. Meanwhile, we also access this data from MM5 model [28] simulation except for the LIDAR. The ground-based wind data (the altitude is about 10 m) are taken from the miniature weather station mounted on the car.

**Figure 6:**
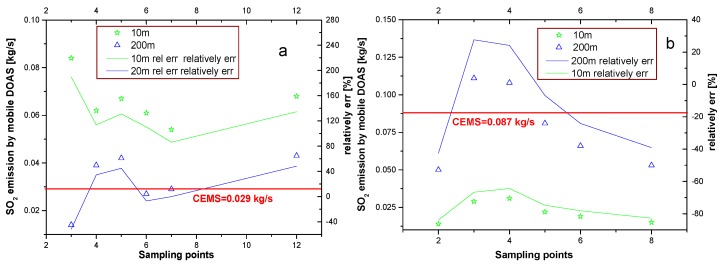
SO_2_ emission and relative deviation. (**a**) SO_2_ emission results on 26 October; (**b**) SO_2_ emission results on 10 November. The dots and lines indicate SO_2_ emission and relative deviation, respectively. The negative relative deviation indicates that the mobile DOAS result is lower than that of CEMS.

The SO_2_ emission is estimated with ground-based wind and plume-height wind for different sampling as discussed in Section 3.1 and compared with CEMS results. Figure 6 demonstrates two examples of SO_2_ emission on 26 October and 10 November under different conditions.

The emission result is closer to that of CEMS when it calculates the emission with 200 m height wind. In this case, the 200 m height is the source from the stack height of 180 m. In addition, further results suggest the difference between mobile DOAS and CEMS, which is lower when the 200 m height wind and driving speed of 30–40 km/h are used to calculate emission.

### 3.3. Comparison of SO_2_ Emission Using Different Wind Data

As discussed in Section 3.2, the SO_2_ emission estimation using mobile DOAS is more accurate when the plume height wind is adopted. The plume height wind is mainly sourced from sounding balloon, model simulation, and wind LIDAR. However, these data rather than ground-based wind data for actual measurement in general are difficult to access. To evaluate the SO_2_ emission error caused by wind, the ground-based wind, 200 m height wind from wind LIDAR or model, and 200 m height wind calculation from ground-based wind are used to estimate emission, and the detailed error analysis for the three types of wind data are performed in this study.

The empirical formula [29] of wind profile shows that the relationship of ground-based wind speed and different altitudes of wind speed, with altitude *z* ≤ 200 m, is as follows:

(10) $$u=u_{0}\cdot {\left(\frac{z}{z_{0}}\right)}^{m}$$

where *z*_0_ is the altitude of the ground-based weather station (*z*_0_ = 10 m), *u*_0_ is the wind speed at the altitude of *z*_0_, and *m* is a factor that relates to atmospheric stability with *m* = 0.15 [29] for “D-class” and *m* = 0.10 for “C-class”. This “C- or D-class” sources from six Pasquill–Turner stability class are derived from the combination of wind speed, solar radiation, and cloud cover: from 1 or A for extremely unstable to 6 or F for extremely stable conditions [30,31]. The atmospheric stability of “D-class” is selected for the majority of measurement time (16, 27 and 29 October and 6, 10, 11 and 13 November), except for 26 October and 28 October, which are regarded as “C-class.” The related classifications are discussed in our previous study, which takes into account wind speed, solar radiation, and cloud cover [28].

**Figure 7:**
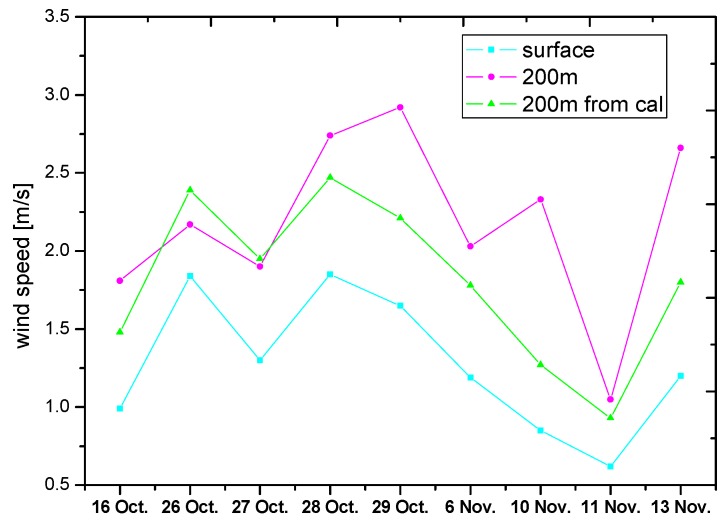
Three types of wind speed. Blue indicates the wind speed source from ground-based wind; pink indicates the wind speed source from 200 m height (LIDAR or model); and green indicates the wind speed source from 200 m height with calculation from ground-based wind.

Figure 7 shows the ground-based wind speed (referred to as A1), 200 m height wind speed source from wind LIDAR or model (referred to as B1), and 200 m height wind speed with calculation from ground-based wind (referred to as C1). The wind speed of A1 is lower than those of B1 and C1. Apart from the wind speed on 10 November and 13 November with the largest difference of 1.06 m/s and 0.86 m/s, respectively, C1 agrees with B1 in most of the conditions. In addition, the wind speed trend is in good agreement for A1, B1, and C1, except for 29 October and 10 November.

**Figure 8:**
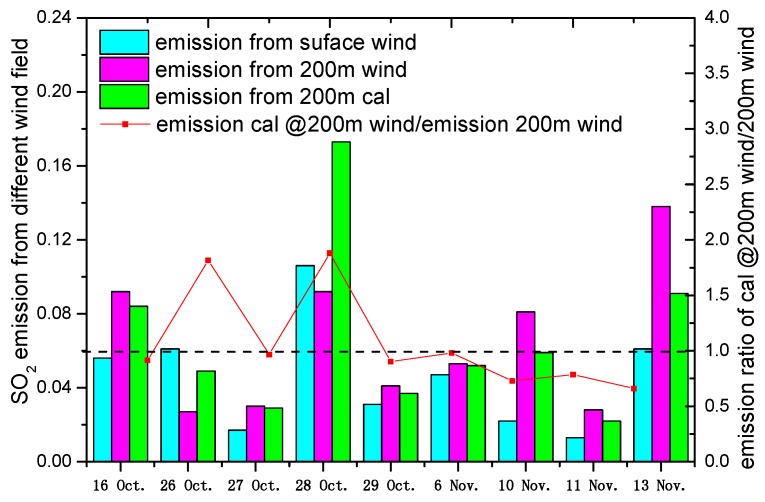
SO_2_ emission estimation with different types of wind and ratios of emission with C1 and B1 wind.

Figure 8 shows that the SO_2_ emission estimations with B1 and C1 winds are larger than those with A1 as presented in Figure 6 in majority of the cases. However, the SO_2_ emission with A1 wind is larger than those with B1 and C1 on 26 October and 28 October because of the deviation of wind direction between ground-based wind and 200 m altitude. The ratios of emission estimation with C1 and B1 winds fluctuate around 1, whereas the maximum ratio is 1.81 and 1.88 on 26 October and 28 October, respectively. This scenario can be explained by the fact that we also consider the wind direction more than wind speed when estimating the emission. However, the empirical formula as presented in Equation (10) reveals that the wind speed at an altitude of 200 m and the hypothesis of wind direction for C1 and A1 agree are prerequisites when calculating the emission with C1 wind. In other words, the significant difference of wind direction between B1 wind and C1 wind can result in large deviation in emission estimation using B1 and C1 winds.

Table 1 lists the SO_2_ emission from mobile DOAS observations with three different wind and CEMS observations. It shows that the similar trends of relative deviation between CEMS and SO_2_ estimation with B1 and C1 winds based on mobile DOAS are presented apart from the results of 26 October and 28 October.

However, although the emission estimation with B1 and C1 winds has a small difference, their ratio fluctuates around 1, which can be explained by the errors of empirical formula and the value of m. In addition, the relatively low deviations for emission estimation with the wind of 200 m height further verify the discussions in Section 3.1. The result of the relatively larger deviations is caused by the fog weather on 29 October. If the plume height wind is difficult to access, then we could estimate emission with the wind calculation from ground-based wind using the empirical formula based on the mobile DOAS observations when the weather system is relatively stable and when vertical wind direction has no significant variation.

### 3.4. Total Errors of Emission Flux

According to the above analysis, the relative deviations of emission estimation are lower under a drive velocity of 30–40 km/h, and the wind field at plume height is selected when flux is calculated. However, the total errors of mobile DOAS have not been listed. This section discusses the total errors of emission flux based on the mobile DOAS observations. The total errors of emission flux can be calculated using Equation (11):

(11) $$\frac{\Delta F_{i}}{F_{i}}=\sqrt{{\left(\frac{\Delta \vec{W}}{\vec{W}}\right)}^{2}+{\left(\frac{\Delta SCD}{SCD}\right)}^{2}+{\left(\frac{\Delta AMF}{AMF}\right)}^{2}+{\left(\frac{\Delta s}{s}\right)}^{2}}$$

From the above formula, four components are included in emission estimation errors: wind error ($∆\vec{W}$), SCD (Δ*SCD*) error, AMF (Δ*AMF*) error, and distance difference (Δ*s*, represented by drive velocity) error. The accuracy of drive speed is about 1% resulting from GPS. The drive speed variation is 1 km/h, and the flux can change by 2% to 3% from the actual calculation. Thus, the variation of 0.3–0.4 km/h (the optimal speed is 30–40 km/h) for drive velocity can result in a change in approximately 1% on emission estimation. As a result, the distance difference error is 1%. In addition, the SCD errors of SO_2_ and NO_2_ are low at 20% and 15%, respectively, and the AMF error of NO_2_ is about 6%. According to the discussions in Section 2.4, the AMF error of SO_2_ is negligible during the measurement periods. Furthermore, the largest error source of emission estimation is from wind field, including wind direction and wind speed errors as listed in Table 2. The average wind error is about 25%, considering the uncertainties of wind direction and wind speed. As a result, the total estimation errors of SO_2_ and NO_2_ are 32% and 30%, respectively.

### 3.5. Emission Estimation of NOx

From Equation (6), the prerequisite for estimation of NOx emission is the knowledge of the ratio of NO and NO_2_, which is expected to be established for ozone-rich condition. However, the measurements are performed close to the stacks of power plants. The reaction of NO with ozone will eventually consume all available ozone, which prevents further conversion of NO into NO_2_. Only after additional ozone-rich air is mixed with the ozone-depleted air masses can the ratio be established. Thus, we analyze the NO, NO_2_, and O_3_ concentration from point instrument observations located 1.7 km away from the power plant during the measurement period of this study. We calculate the ratio of NO and NO_2_ (Table 3) under an O_3_ concentration higher than NO concentration. Moreover, the NO_x_ lifetime factor is calculated using wind speed with Equation (8).

**Figure 9:**
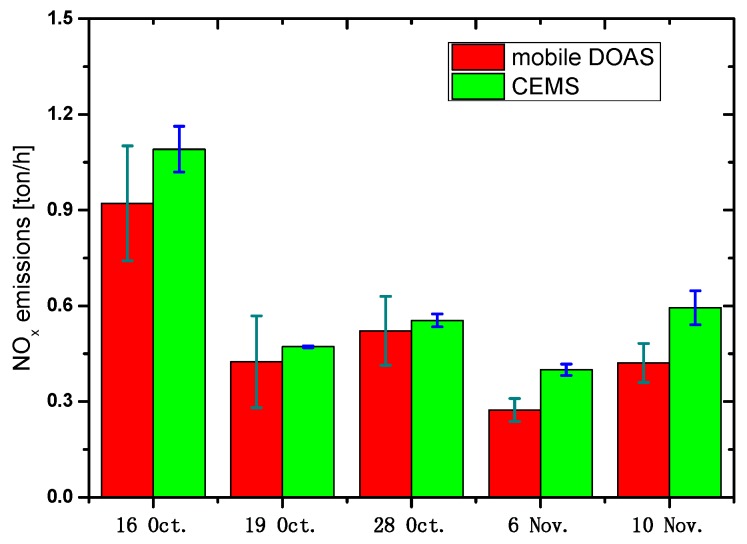
NO_x_ emission flux from mobile DOAS and CEMS observations.

The NOx emission is then estimated with *R, C_L_*, and NO_2_ emission flux (from mobile DOAS observation) based on Equation (6). Figure 9 shows the comparison of NO_x_ emission between mobile DOAS and CEMS, indicating a good agreement with the average flux of 0.15 ± 0.06 kg/s and 0.17 ± 0.07 kg/s from mobile DOAS and CEMS observations, respectively.

## 4. Conclusions

A power plant in Shijiazhuang city was selected as the experimental site to study the emission flux estimation error with a mobile DOAS system under different measurement conditions and is explored to detect NO_x_ flux from power plant emission. This study has significantly contributed to the measurement of mobile DOAS on emission from air pollution sources, thus improving estimation accuracy.

The optimal drive velocity of 30–40 km/h and the wind field at plume height are selected when mobile DOAS observations are made by comparing the SO_2_ emission flux from mobile DOAS with CEMS under different drive speeds and wind fields (ground-based and plume-height wind field). The emission flux can also be estimated with the plume-height wind speed based on empirical formula calculation when the weather system is relatively stable and vertical wind direction has no significant variation. In addition, the total error of mobile DOAS observations is estimated, which are sourced from wind field error, VCD error, and drive speed variation error. As a result, the total errors of SO_2_ and NO_2_ are 32% and 30%, respectively, taking into account wind field uncertainty of 25%, SO_2_ SCD uncertainty of 20%, NO_2_ SCD uncertainty of 15%, NO_2_ AMF uncertainty of 6%, and drive speed uncertainty of 1%. Finally, the NOx emission from the power plant is estimated with a value of 0.15 ± 0.06 kg/s, which is in good agreement with that from CEMS observation with a value of 0.17 ± 0.07 kg/s.

## Figures and Tables

**Table 1:** Comparison of SO_2_ emission between mobile DOAS for three different winds and CEMS. The percentage indicates the deviation with respect to emission from CEMS. The negative values imply that CEMS is lower than those from mobile DOAS; the positive values imply otherwise.

	Emission from CEMS kg/s	Surface Wind %	200 m Wind %	Calculation at 200 m Wind %
16 October	0.096	−41.67	−4.17	−12.50
26 October	0.029	110.34	−6.90	68.97
27 October	0.042	−59.52	−28.57	−30.95
28 October	0.069	53.62	33.33	150.72
29 October	0.139	−77.70	−70.50	−73.38
6 November	0.064	−26.56	−17.19	−18.75
10 November	0.087	−74.71	−6.90	−32.18
11 November	0.056	−76.79	−50.00	−60.71
13 November	0.119	−48.74	−15.97	−23.53

**Table 2:** Wind field uncertainty of emission estimation.

Date	Time	Wind Direction (Degree)	Wind Speed (m/s)	Uncertainties from Wind Direction	Uncertainties from Wind Speed	Uncertainties from Wind
16 October	10:00–12:00	274.13 ± 2.15	1.81 ± 0.4	1%	22%	22%
26 October	12:30–14:00	135.25 ± 9.51	2.17 ± 0.2	14%	10%	17%
27 October	12:00–13:00	123 ± 6	1.9 ± 0.3	19%	14%	24%
28 October	11:40–13:00	183.02 ± 10.10	2.74 ± 0.42	2%	15%	16%
29 October	13:30–14:10	112.21 ± 1.81	2.92 ± 0.36	10%	12%	16%
6 November	11:00–13:00	230.8 ± 12	2.03 ± 0.64	24%	33%	41%
10 November	13:00–14:30	167.67 ± 9.46	2.33 ± 0.54	3%	24%	25%
11 November	11:30–12:10	211.17 ± 28.06	1.05 ± 0.29	33%	29%	44%
13 November	11:40–12:10	192.58 ± 7.29	2.66 ± 0.61	3%	23%	24%

**Table 3:** NO, NO_2_, and O_3_ concentration and the values of *R* and *C_L_*.

Date	NO mg/m^3^	NO_2_ mg/m^3^	Leighton Ratio	O_3_ mg/m^3^	*R*	*C_L_*	Wind Speed m/s
16 October	0.041	0.072	0.57	0.042	1.57	1.06	1.81
19 October	0.014	0.041	0.34	0.024	1.34	1.04	3.00
28 October	0.04	0.116	0.34	0.048	1.34	1.06	2.74
6 November	0.015	0.044	0.34	0.048	1.34	1.07	2.03
10 November	0.028	0.063	0.44	0.028	1.44	1.05	2.33
